# Risk factors of pediatric steroid-induced ocular hypertension

**DOI:** 10.1007/s00417-024-06669-6

**Published:** 2024-10-26

**Authors:** Fumio Takano, Kaori Ueda, Yuko Yamada-Nakanishi, Makoto Nakamura

**Affiliations:** Division of Ophthalmology, Department of Surgery, Kobe University Graduate School of Medicine, 7-5-2, Kusunoki-cho, Chuo-ku, Kobe, 650-0017 Japan

**Keywords:** Steroid-induced ocular hypertension, Risk factor, Systematic steroid administration

## Abstract

**Purpose:**

Steroid-induced ocular hypertension (SIOH) is a significant ocular complication of pediatric steroid administration. In this study, we analyzed the risk factors associated with pediatric SIOH.

**Methods:**

We retrospectively collected data from 78 children under 20 years of age who received systemic steroids during hospitalization. The data included age, gender, primary disease, intraocular pressure (IOP) before and one month after administration, total monthly steroid dose adjusted for body weight (BW), and one-month changes in red blood cell, white blood cell, and platelet counts. A multivariate analysis was used to identify risk factors related to steroid responsiveness.

**Results:**

Thirty patients (38.5%) were classified as steroid responders, and 48 as non-responders. The median IOP during the first month of steroid treatment was 24.0 mmHg (IQR; 23.0–28.3) for responders and 15.0 mmHg (IQR; 12.3–18.0) for non-responders. The Generalized Estimating Equations analysis revealed that younger age, male sex, primary disease, increase the amount of white blood cell (WBC) and total steroid dose per BW in one month were independently associated variables. The receiver operating characteristic analysis also revealed that the cutoff values for age, total monthly steroid dose, the increase amount of WBC were 11.0 years, 40.7 mg/kg and 3.40 × 10²/µl respectively.

**Conclusion:**

High-dose steroid administration, especially in male, younger patients, necessitates careful monitoring for IOP changes during treatment. WBC count also needs to be monitored during IOP follow-ups.

**Key messages:**

***What is known***

- Steroid-induced ocular hypertension (SIOH) is one of the essential complications during steroid administration, but only limited analyses have been performed in children.

***What is new***

- A comprehensive analysis of multiple factors was performed that are predicted to be associated with pediatric SIOH from previous literature.
- Younger age, male sex, primary disease, increase the amount of WBC, and higher total monthly steroid dose were extracted as risk factors of SIOH.
- This study can contribute to the prediction of cases in which ophthalmologic examinations are particularly important during systemic steroid administration in children.

## Introduction

Steroid-induced ocular hypertension (SIOH) is a common side effect of steroid administration. This complication can occur at any age and is a risk associated with all administration methods, including topical, intranasal, inhalation, oral, and injection [1, 2].

If left untreated, SIOH can progress to steroid-induced glaucoma (SIG), a serious steroid-induced ophthalmic complication that can result in permanent loss of vision. Furthermore, because SIOH frequently presents with few symptoms, regular ophthalmologic examinations are recommended during steroid treatment.

The amount and duration of steroid use that can result in SIOH varies by case. Intraocular pressure (IOP) elevations typically occur several weeks after steroid administration [3–5], and are dose-dependent manner [1, 6].

Steroid responders are patients who exhibit an increase in IOP in response to steroids. In adults, numerous studies have identified risk factors associated with steroid responders, such as a history of glaucoma, advanced age, and certain systemic disorders [1]. However, the risk factors for SIOH in pediatric cases have not been thoroughly studied. Although some studies suggest that younger age, a family history of glaucoma, or certain types of steroids are independently associated with SIOH in children [7], most studies including prospective studies provide incomplete results with a small number of cases or limited disease coverage.

This study sought to identify risk factors for steroid responsiveness in children who had received systemic steroid therapy.

## Materials and methods

The Institutional Review Board of the University Hospital authorized this retrospective observational study (No. B230085). The study protocol followed the Declaration of Helsinki. Informed consent was waived; instead, patients could review study information on the hospital’s website and withdraw consent at any time as an opt-out option.

The study included hospitalized children under the age of 20 years who received systemic steroid treatment (oral or intravenous administration) and had their IOP measured before and after treatment at Kobe University Hospital between January 2016 and November 2023. No patients were given antiglaucoma medications during the study period. We collected primary clinical data from all patients, including gender, age, body weight (BW), the primary disease category requiring steroid treatment (blood disease, renal disease, central nervous system disease, and connective tissue disease), the total monthly steroid dose (mg/kg), and changes in red blood cell (RBC), white blood cell (WBC), and platelet (Plt) counts before and after steroid administration. All administered steroids were analyzed using their prednisone equivalents. IOP was measured using an iCare IC100 tonometer (M.T. Technica, Co., Ltd., Tokyo, Japan) before and one month after steroid treatment. Because Steroid responders were defined as patients with an elevation in IOP of ≥ 21 mmHg or an increased amount of IOP ≥ 6 mmHg in either the left or right eye, similar to the criteria used for adults [8].

Continuous variables were reported as median and interquartile range. The explanatory variables used for the binomial logistic analysis were age, gender, BW, blood count differences before and after steroid treatment, and total monthly steroid dose per BW. Considering confounding in both eyes, Generalized Estimating Equations (GEE) model was used to analyse risk factors. The EZR software (http://www.jichi.ac.jp/saitama-sct/SaitamaHP.files/download.html) was used to perform various statistical analyses, such as Fisher’s exact test, Mann–Whitney U test, GEE analysis, and receiver operating characteristic (ROC) analysis. The results were considered statistically significant at *p* < 0.05.

## Results

The study had 30 responders (38.5%) and 48 non-responders. The characteristics of the patients was summarized in Table 1. The median IOP during the first month of steroid administration was 23.0 mmHg (IQR; 21.0–26.3) in right eye and 23.5 mmHg (IQR; 23.0–28.3) in left eye for responders, and 16.0 mmHg (IQR; 14.0–18.0) in right eye and 15.0 mmHg (IQR; 12.0–18.0) in left eye for non-responders. Univariate analysis found significant differences in patient age, BW, primary disease, and total monthly steroid dose per BW (*p* < 0.01) between the two groups.

**Table 1:** Patient background

Variable	Non-responder	Responder	*p*-value
Number of patients	48	30	
male (%)	50	71.4	0.102^a^
age (IQR, yo)	13.5 (9.0, 16.0)	7.5 (3.0, 11.0)	< 0.001^b^
BW (IQR, kg)	44.1 (29.3, 56.5)	25.1 (16.1, 35.5)	< 0.001^b^
disease category			0.021^a^
hematologic disease	13	11	
renal disease	10	13	
central nervous system disease	4	2	
connective tissue disease	21	4	
Amount of prednisolone (IQR, mg/kg BW/month)	32.7 (18.9, 56.7)	59.3 (49.4, 76.1)	< 0.001^b^
Increase amount of RBC (IQR, ×10⁴/µl)	0.015 (-0.33, 0.46)	4.8 (3.8, 5.1)	0.558^b^
Increase amount of WBC (IQR, ×10²/µl)	1.75 (-0.65, 4.78)	4.6 (0.03, 10.2)	0.093^b^
Increase amount of PLT (IQR, ×10⁴/µl)	5.5 (-58.3, 62.8)	17 (-29.3, 113.5)	0.391^b^
pretreatment IOP_R (IQR, mmHg)	15.0 (14.0, 17.0)	15.0 (12.0, 17.0)	0.429^b^
posttreatment IOP_R (IQR, mmHg)	16.0 (14.0, 18.0)	23.0 (21.0, 26.3)	< 0.001^b^
pretreatment IOP_L (IQR, mmHg)	15.0 (14.0, 17.0)	14.0 (12.0, 17.0)	0.238^b^
posttreatment IOP_L (IQR, mmHg)	15.0 (12.0, 18.0)	23.5 (23.0, 28.3)	< 0.001^b^

yo; years old, IQR; interquartile range, BW; body weight, ^a^ Fisher’s exact test, ^b^ Mann–Whitney U test

Table 2 shows the result of GEE analysis. Younger age, male sex, primary disease, increase the amount of WBC, and higher total monthly steroid dose were independent risk factors.

**Table 2:** Results of GEE analysis

	estimate	san.se	wald	*p*
(Intercept)	1.404282	0.554835	6.405921	0.011374
age	-0.12903	0.040185	10.3098	0.001323
sex	-1.18345	0.47293	6.261902	0.012336
category	-0.6414	0.235236	7.434472	0.006399
Amount of prednisolone	0.010767	0.003848	7.828566	0.005143
Increase amount of WBC	0.101213	0.036756	7.582548	0.005894
Increase amount of RBC	-0.24185	0.242252	0.996662	0.31812
Increase amount of PLT	-0.00166	0.002059	0.651456	0.419593

**Fig. 1:**
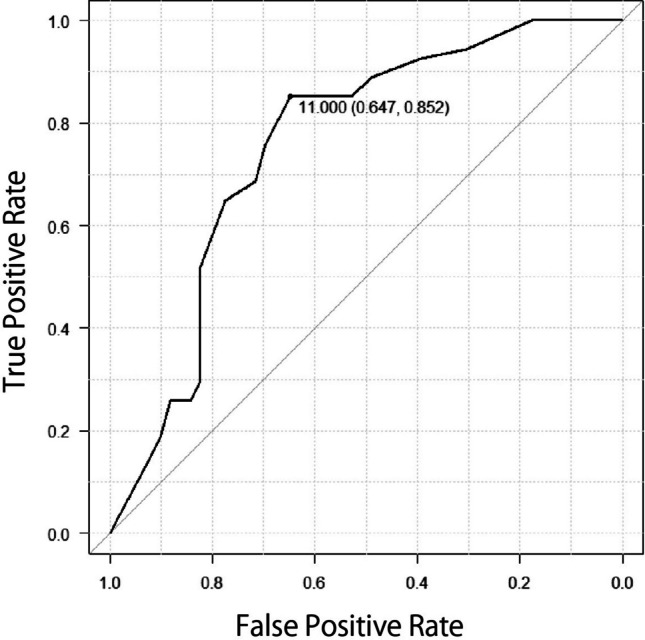
ROC curve based on the ages and responder AUC; 0.752, 95% CI; 0.641–0.863

**Fig. 2:**
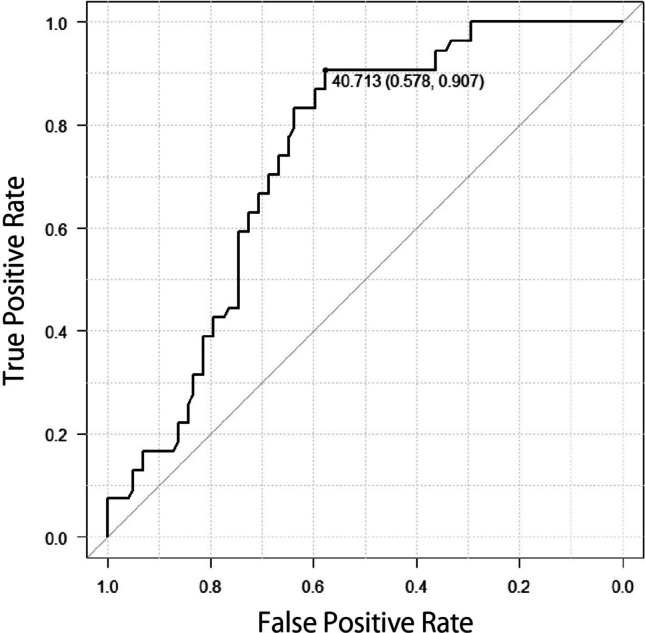
ROC curve based on the total steroid volume (/kg BW/month) and steroid responder AUC; 0.738, 95%CI; 0.628–0.848

**Fig. 3:**
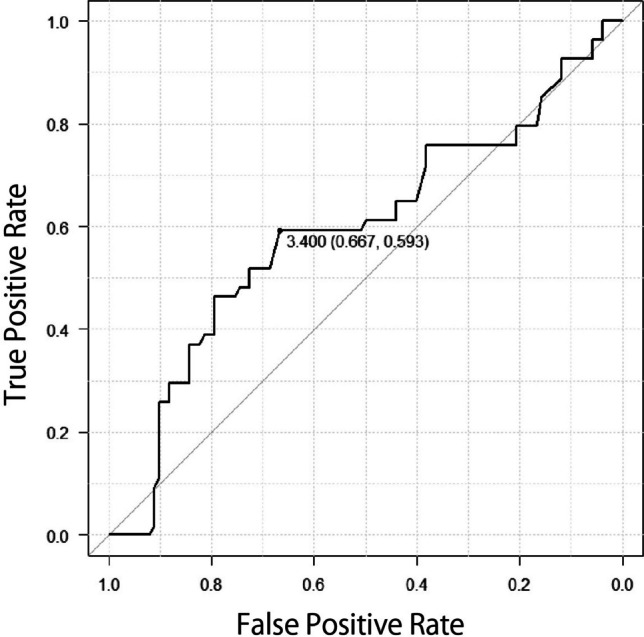
ROC curve based on the amount of increase in leukocytes and steroid responder

Figure 1 shows the ROC curve for age, with a cutoff value of 11.0 years (area under the curve (AUC); 0.757, 95%CI; 0.68–0.833). Figure 2 depicts the ROC curve for total steroid dose, with a cutoff value of 40.7 mg/kg BW/month (AUC; 0.738, 95%CI; 0.661–0.815). Figure 3 is also ROC curve for the increase of about in WBC in one month, with a cutoff value of 3.40 × 10²/µl (AUC; 0.594, 95%CI; 0.495–0.692).

## Discussion

This retrospective study included more cases than those in previous studies and investigated risk factors in a diverse group of pediatric steroid responders. Young age, male sex, primary disease, increase amount of WBC, and high steroid doses were discovered to be independently associated with SIOH.

Krag et al. hypothesized that young age is a risk factor for pediatric steroid responders [9]. Some studies have suggested a link between age and high IOP, but the relationship is still debated. According to Gupta et al., patients younger than 6 years old are more likely to develop SIOH [10]. Sugiyama et al. found that SIOH is more common in children under the age of 10 [6]. 

In this study, steroid responders were younger, with a cutoff age of 11.0 years, which is consistent with some previous findings. Previous studies have shown that glucocorticoids can alter the morphological and biological structure of the trabecular meshwork (TM) and inhibit aqueous humor outflow, resulting in increased IOP [1, 11]. It has also been suggested that the TM continues to grow until around the age of 8 [12], implying that steroids may have a greater impact on the immature TM in younger patients.

Rodrigo et al. investigated gender differences in SIOH using a SIG rat model, discovering that male rats had significantly higher IOP than female rats after a 24-week follow-up period [13]. A clinical retrospective analysis of 1783 cases of SIOH following photorefractive keratectomy identified male sex as a risk factor [14].

Research on TM length may shed some light on the link between the male sex and SIOH. Cho et al. measured the TM length in 26 responders and 78 non-responders and discovered that patients with SIOH had significantly shorter TM than non-responders [15]. Kasuga et al. also found that males had a shorter TM height than females, though the differences were not significant [16]. These findings suggest that males with shorter TMs may be more vulnerable to steroid exposure.

Primary disease was also extracted as one of the risk factors in relation to SIOH. Because there are too many explanatory variables, it is difficult to analyze each disease are included in GEE analysis. We checked for average steroid dose in each category. The amount of steroid use was 59.0, 66.8, 173.0, and 100.6 mg/kg for hematologic, renal, central nervous system, connective tissue disease, respectively, whereas it was 39.6, 72.3, 76.8, 33.7 mg/kg in the non-responder group. In the steroid responder group, steroid dosage is particularly high in central nervous system and connective tissue disease. Therefore, we speculate that these difference by category is probably due to steroid dosage.

The current study’s cutoff steroid dose was 40.7 mg/kg/month, or approximately 1.3 mg/kg/day, indicating a high dose. There is a discussion about the relationship between steroid dose and SIOH. Some studies indicate a dose-dependent relationship [1, 6], whereas others have found no causal link between high steroid doses and SIOH [17–19].

Previous research has focused on children with specific diseases [4, 6, 18]. To identify risk factors for steroid responders, we classified primary diseases requiring steroid treatment. However, our current study did not reveal a clear relationship between primary disease and responders. Only one previous study proposed a link between connective tissue disease and elevated IOP; thus, the role of primary disease in steroid responsiveness is assumed to be minor.

This study examined the difference in blood cell counts before and after steroid administration. The findings indicate that WBC counts may contribute to IOP elevations in children with ALL [5]. Increased hematocrit and blood viscosity are also risk factors for high IOP [20, 21]. In our study, increase the amount of WBC was extracted the risk factor of SIOH as other previous research. However, the cutoff value is unreliable due to low AUC, that is expected to be less important than other risk factors. We hypothesize that in hematologic diseases, blood cell counts frequently fluctuate rapidly during treatment, making it difficult to accurately assess count changes. Although this is just for reference, 18 (60.0%) responders and 15 (31.2%) non-responders had a WBC change greater than the cutoff value of 3.4 × 10²/µl.

There are several limitations to this study. First, we used rebound tonometry to check the IOP in each examination, although applanation tonometry is gold standard device to measure IOP. Because children sometimes refuse applanation tonometry, because of its invasiveness. Moreover, some children undergoing anticancer agent treatment are isolated in private rooms. In such cases, rebound tonometry can be very useful. Therefore, in this study, we decided to standardize the results of IOP measurements to rebound tonometry.

We limited the follow-up period to one month after starting steroid treatment. This is because some patients are discharged from the hospital months after receiving steroids. Subsequently, their ophthalmologic care is managed at their own clinic, making it difficult for us to follow up directly. Consequently, we could not determine whether the duration of steroid administration is a risk factor for steroid responders. To address this issue, a prospective multicenter study is recommended.

For our analysis, we converted all administered steroids to prednisone equivalents to account for differences between the various steroid types used. As a result, we did not investigate the association between the types of steroids used and steroid responders. Given that high-titer dexamethasone has been shown to significantly increase IOP [18, 22], it is also important to consider the type of steroids used.

Furthermore, systemic steroid administration may necessitate large infusion volumes, causing an increase in IOP due to fluid overload [23]. Therefore, the infusion loading volume should be considered for a more thorough analysis.

The definition of SIOH is not a consensus. Several studies divided SIOH into three categories, non-responder, intermediate- responder, high-responder. Because in this study there are few high-responder, and the main point in this study is to search for the risk factor of SIOH, so we decided to follow the papers that divide SIOH into two categories, responder or non-responder. If we have more participants, we will analyze using three categories.

In conclusion, in children receiving systemic steroid administration, age of < 11 years, male gender, primary disease, high increase in WBC during treatment, and high steroid doses may contribute to steroid-induced IOP elevation. Although our findings are reasonably consistent with clinical findings and previous reports, a more precise analysis will require prospective studies involving a large cohort of patients.
